# Importance of intraoperative indocyanine green imaging in the management of non-occlusive mesenteric ischemia: a case report

**DOI:** 10.1186/s40792-023-01614-x

**Published:** 2023-02-27

**Authors:** Ryohei Miyashita, Masato Kitazawa, Shigeo Tokumaru, Satoshi Nakamura, Makoto Koyama, Yuta Yamamoto, Nao Hondo, Satoru Miyazaki, Yuji Soejima

**Affiliations:** Division of Gastroenterological, Hepato-Biliary-Pancreatic, Transplantation and Pediatric Surgery, Department of Surgery, Shinshu University School of Medicine, 3-1-1 Asahi, Matsumoto, Nagano 390-8621 Japan

**Keywords:** Non-obstructive intestinal ischemia, Indocyanine green, Postoperative bleeding

## Abstract

**Background:**

Non-obstructive intestinal ischemia (NOMI) is caused by intestinal vascular spasm and has a poor prognosis if not diagnosed and treated early. Indocyanine green (ICG) fluorescence imaging has been reported to be useful for the intraoperative assessment of the extent of intestinal resection required for NOMI. Few reports have described massive intestinal bleeding after conservative management of NOMI. We report a case of NOMI with massive postoperative bleeding from the site of an ICG contrast defect found before the initial surgery.

**Case presentation:**

A 47-year-old woman with hemodialysis-dependent chronic kidney disease presented complaining of severe abdominal pain. A computed tomography scan showed portal gas and dilation of the small intestine, leading to a diagnosis of NOMI and subsequent emergency surgery. At the time of initial surgery, the contrast effect of ICG was slightly reduced, showing a granular distribution in the ascending colon to cecum (fine grain pattern) and significantly reduced in parts of the terminal ileum except around blood vessels (perivascular pattern). However, there was no obvious gross necrosis of the serosal surface, and the intestinal tract was not resected. The acute postoperative course was uneventful; however, the patient went into shock on the 24th postoperative day due to massive, small intestinal bleeding, and emergency surgery was performed. The bleeding originated from the section of the ileum that had complete loss of ICG contrast effect before the initial surgery. A right hemicolectomy with the terminal ileum resection was performed, and an ileo-transverse anastomosis was performed. The second post-operative course was uneventful.

**Conclusions:**

We report a case of delayed hemorrhage of the ileum shown to have poor blood flow on ICG imaging at the initial surgery. Intraoperative ICG fluorescence imaging is useful in assessing the degree of intestinal ischemia for NOMI. When patients with NOMI are followed up without surgery, complications such as bleeding should be noted.

## Background

Non-occlusive mesenteric ischemia [NOMI) is caused by intestinal ischemia due to spasms of peripheral intestinal vessels without fixed vascular stenosis or occlusion [1]. Risk factors include age, dialysis, cardiac disease, and cardiovascular surgery. Dehydration, together with these risk factors, is often a precipitant. It is associated with high mortality and a poor prognosis [2]. Its treatment requires correction of dehydration and continuous administration of vasodilators in the absence of- and bowel resection in the presence of intestinal necrosis. In NOMI, the ischemic areas are sparsely distributed, and the disease sometimes progresses rapidly; therefore, rapid and accurate diagnosis and treatment decisions are necessary. The extent of bowel resection is sometimes difficult to determine by gross findings. Bulkley reported that intraoperative fluorescein fluorescence patterns can assess intestinal viability [3]. And recently, Nitori [4] reported the usefulness of indocyanine green (ICG) imaging for intraoperative intestinal blood flow assessment in NOMI. There are few reports on the delayed complications of NOMI. We report a case of delayed hemorrhage from the unresected ileum in an area of poor blood flow previously detected by ICG fluorescence imaging, although there was no obvious necrosis on the serosal surface during intraoperative inspection.

## Case presentation

**Fig. 1:**
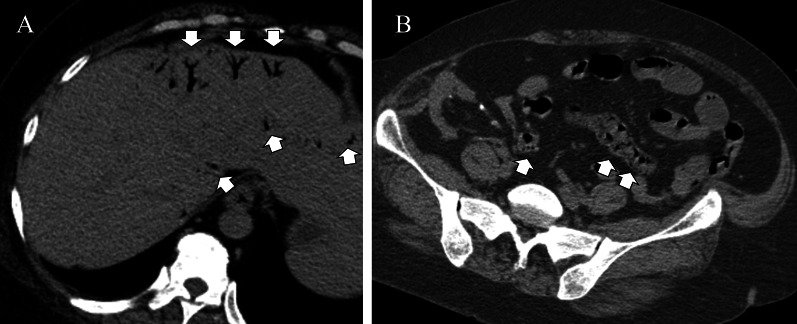
**A** Plain abdominal CT reveals portal gas (arrows). **B** CT also showed emphysema in the small intestine (arrows) and ascending colon

**Fig. 2:**
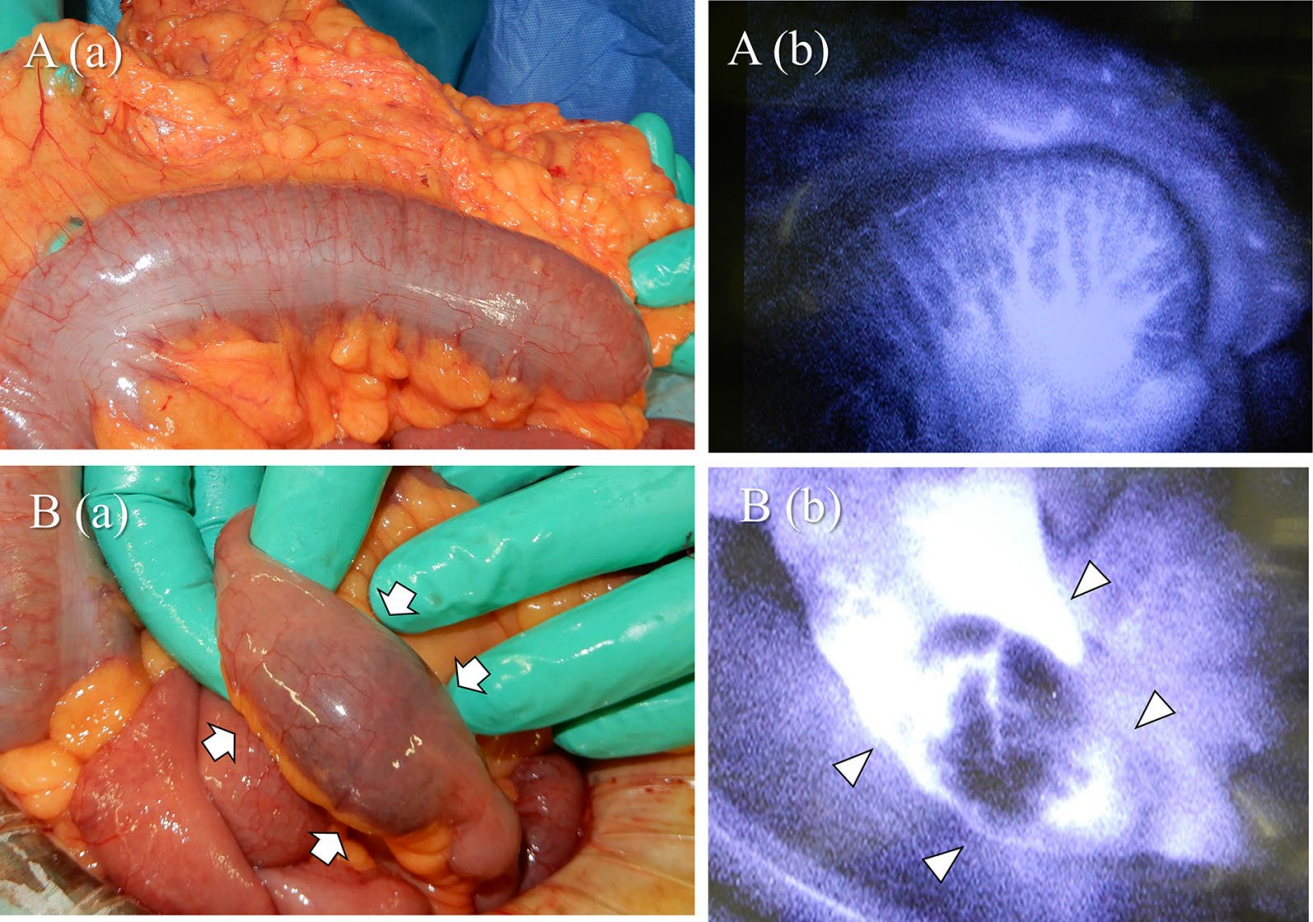
**A** (**a**) In the ascending to cecum, no changes in the serosal surface were observed with the naked eye. (**b**) Intraoperative ICG image showed a granular distribution of fluorescent dye, indicating “fine granular pattern”. **B** (**a**) Intraoperative findings showed no obvious intestinal necrosis. However, there was an area of dark red serosa at the terminal ileum (arrows). (**b**) ICG fluorescence was deficient except in perivascular area, indicating “perivascular pattern” (arrowheads)

**Fig. 3:**
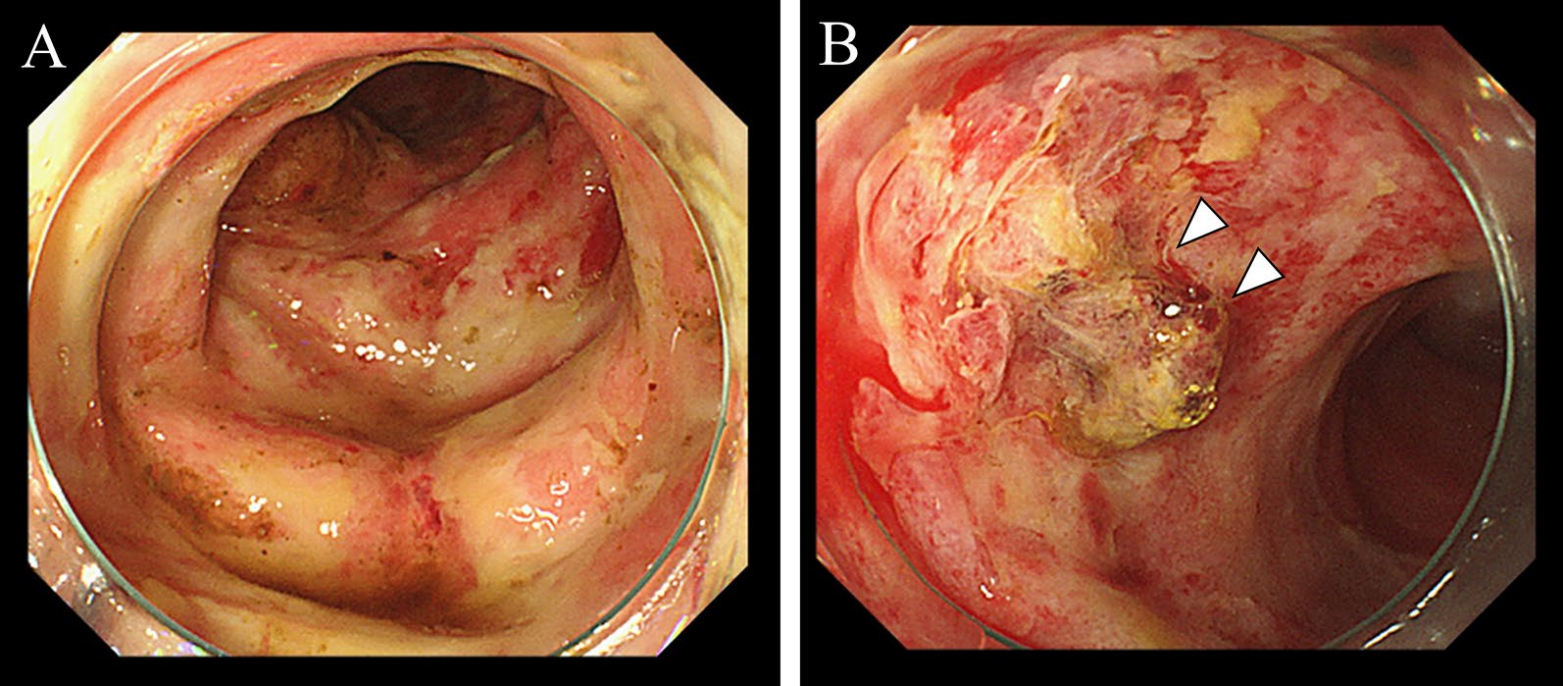
**A** Emergency colonoscopy revealed extensive mucosal erosions in the ascending colon to cecum. **B** Deep ulcer with a exposed blood vessel were observed in the terminal ileum (arrowheads)

**Fig. 4:**
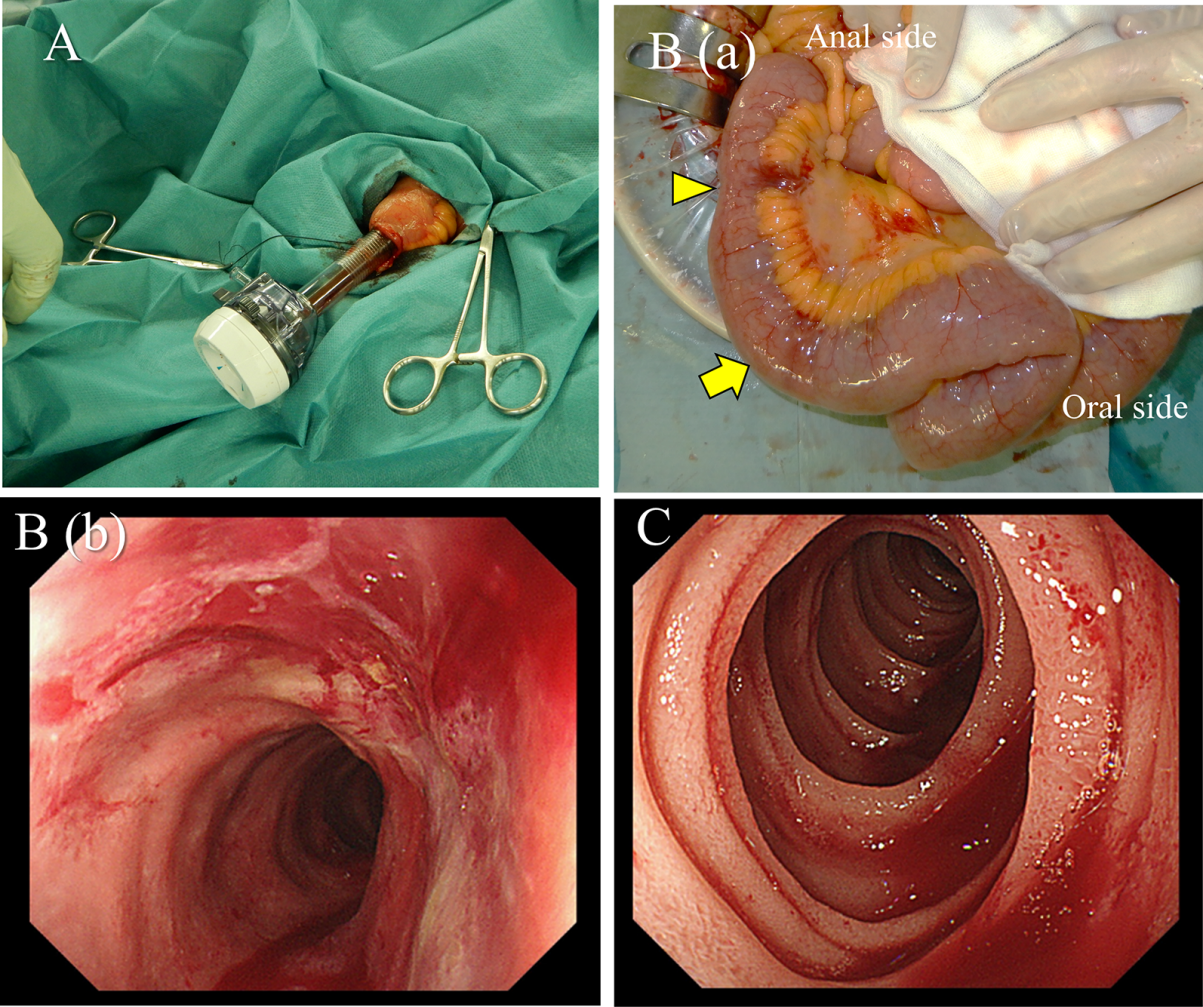
**A** Intraoperative endoscopy was performed during the second surgery. **B** (**a**) Intraoperative findings showed no obvious intestinal necrosis, but the terminal ileum wall on the 25 cm oral side from the ileocecal valve was hard and thickened (arrowhead). A laparoscopic trocar was inserted 5 cm orally from there (arrow). (**b**) Intraoperative endoscopic observation on the anal side confirmed the presence of the deepest longitudinal ulcer in the terminal ileum, where the bleeding site was expected. **C** Observation of the oral small intestine revealed no ulcerative lesions that could have caused the hemorrhage

**Fig. 5:**
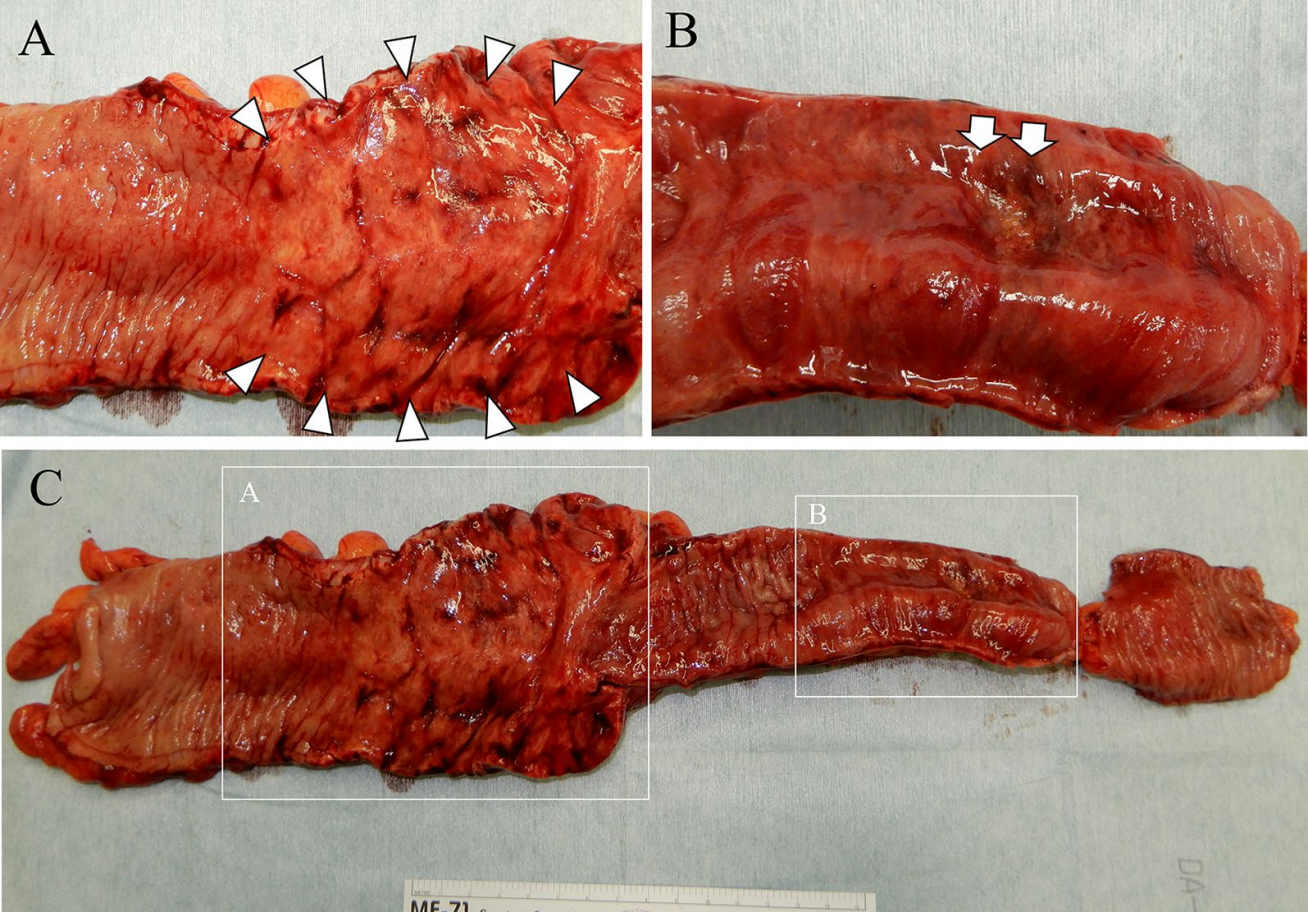
**A** Extensive mucosal erosions were found in the ascending colon to cecum (arrowhead), same lesion in Figs. 2A and 3A. **B** Deep longitudinal ulceration were observed in the terminal ileum (arrows), same lesion as in Figs. 2B, 3B and 4B. **C** A 30 cm terminal ileum with a hemorrhagic ulcer lesion and a 20 cm cecum to ascending colon with erosions were resected together

A 47-year-old woman was transferred to our hospital complaining of abdominal pain. The pain was severe and accompanied by a loss of consciousness. She was known with chronic kidney disease and was on regular hemodialysis. On arrival at the hospital, her blood pressure was 93/67 mmHg, pulse 78 beats per minute, body temperature 36.7 °C, and Glasgow Coma Scale score of 13/15 (E3V4M6), with loss of consciousness. She had severe tenderness over the entire abdomen without rebound or guarding. An arterial blood gas analysis on room air showed a pH of 7.535, pCO2 of 28.1 mmHg, pO2 of 107 mmHg, HCO3 of 23.6 mmHg, base excess of − 5.1, and lactate of 10 mg/dl. Blood tests showed a mildly elevated white blood cell count of 10,950/μl, urea of 27.9 mg/dl, and creatinine of 5.59 g/dl, which was high for a dialysis patient, creatine kinase of 67 U/L, and C-reactive protein of 0.38 which was almost normal. Computed tomography (CT) revealed extensive portal vein gas (Fig. 1A) and intestinal emphysema in the ileum and ascending colon. (Fig. 1B). The patient was diagnosed with NOMI and underwent emergency surgery. There was no intestinal fluid contamination in the abdominal cavity, but there was a moderate accumulation of serous ascites. The serosa of the ascending colon to cecum [Fig. 2A(a)] and terminal ileum [Fig. 2B(a)] were red in areas, but there was no obvious necrosis. Intraoperative ICG imaging with 5 mg intravenous ICG administration showed a granular distribution of the fluorescent dye (fine granular pattern) in the ascending colon to cecum [Fig. 2A(b)] and a poorly fluorescent lesion except around blood vessels (perivascular pattern) in the terminal ileum [Fig. 2B(b)] [3]. Because the serosal side of the intestine was not grossly necrotic, we decided to manage the patient conservatively and only perform a second-look surgery if there were clinical, blood, and CT findings suggestive of necrosis the next day, and so bowel resection was not performed. Postoperatively, prostaglandin E1 was injected intravenously at 0.05 ug/kg/min. A contrast-enhanced CT performed the day after the surgery showed good blood flow in the ascending colon and ileum, and abdominal findings and blood tests had not worsened; therefore, a second-look surgery was not performed. Intraoperatively, the intestinal emphysema disappeared and the serous membrane color tended to improve, so postoperative open abdomen management was not performed and the abdomen was closed by conventionally. The acute postoperative course was uneventful; however, the patient required prolonged hospitalization for diabetes control. On POD 24, the patient suddenly passed a large quantity of bloody stool. An emergency colonoscopy revealed a longitudinal ulcer in the ascending colon to cecum (Fig. 3A), and a deep ulcer, with exposed blood vessels, in the terminal ileum (Fig. 3B). However, since there was no active bleeding, we decided to treat the patient conservatively with red blood cell transfusion alone, without emergency endoscopic treatment or surgery. On POD25, a large amount of blood was again found in her stool, and she went into shock. She received a massive blood transfusion and underwent an emergency laparotomy. The patient was in a state of shock and required urgent treatment. Although intravenous radiology was a treatment option, hemostasis by laparotomy was chosen, because the CT and endoscopy of the previous day showed that the bleeding site was in the terminal ileum, hemostasis by surgery was easy, and the patient was ready for surgery quickly. A large amount of blood in the colon and small intestine was seen through the serous membrane, suggesting a severe bleed. The terminal ileum wall 25 cm from the ileocecal valve was firm and thickened, but there were no obvious changes on the serous surface of the rest of the small intestine [Fig. 4B (a)]. The thickened terminal ileum was the most likely site of bleeding, and the mesenteric vessels in the area were first ligated to stop bleeding. Next, an incision was made in the terminal ileum, and intraoperative endoscopy was performed (Fig. 4A). This confirmed the presence of a deep longitudinal ulcer in the terminal ileum, where the origin of the bleeding was expected to be located [Fig. 4B(b)]. The extent of intestinal resection was determined after confirming that ulceration was present only in the terminal ileum and ascending colon to the cecum, with no ulcerative lesions on the oral side from the deep longitudinal ulcer in the terminal ileum (Fig. 4C). Right hemicolectomy including the terminal ileum of the bleeding ulcer lesion was performed and an ileo-transverse colon anastomosis was performed by hand. The resected specimen showed extensive mucosal erosion of the ascending colon to cecum (Fig. 5A) and a deep longitudinal ulcer with exposed vessels in the terminal ileum (Fig. 5B). Thirty cm of the terminal ileum and 20 cm of the ascending colon to cecum was resected (Fig. 5C). Her postoperative course after the second surgery was uneventful, and she was discharged on the 28th day after the second surgery. One year after the surgery, NOMI, and intestinal bleeding had not recurred.

## Discussion

NOMI is a disease with a poor prognosis, in which vasospasm causes ischemia and necrosis of the intestinal tract despite the absence of organic obstruction of the intestinal feeding vessels. Risk factors include age, dialysis, post-cardiovascular surgery, and cardiac disease [5, 6]. The mortality rate ranges from 56 to 79% [7]. If the intestinal tract is not necrotic, correction of dehydration, discontinuation of vasoconstrictors, and a continuous infusion of papaverine hydrochloride, prostaglandin E1 (PGE1), and nitroglycerin from the superior mesenteric artery (SMA) are considered effective treatments [8, 9]. Recently, the efficacy of intravenous PGE1 has been reported [10], and it has been considered an alternative to continuous intra-arterial infusion therapy through the SMA. However, if the intestine is irreversibly necrotic, surgical resection is necessary. NOMI is characterized by a discontinuous and segmental distribution of the necrotic intestine [11]. It is difficult to determine the extent of the necrotic intestine and the degree of ischemia only by gross observation from the serous side. Our indication for bowel resection is only when there is obvious necrosis or suspected ischemia, but the resection is small. Otherwise, second-look surgery is performed or a contrast-enhanced CT scan is performed within 12 h after surgery to consider the indication for reoperation. ICG fluorescence has been reported to be useful in diagnosing intestinal ischemia, which is difficult to diagnose with the naked eye and may be useful in determining the required extent of resection [2]. In this case, the ascending colon, which showed a fine granular ICG fluorescence pattern, had extensive longitudinal ulceration. The site of deep longitudinal ulceration at the end of the ileum showed areas of ICG fluorescence only around the vessels, while other areas lacked ICG fluorescence (perivascular pattern) [3]. These lesions, suggestive of decreased blood flow, were not resected at the first surgery, because only erythematous changes on the serosal surface and no obvious necrosis were observed. Delayed intestinal bleeding after NOMI, as in this case, is extremely rare, and there have been no similar reports in the past. In this case, intestinal bleeding was a delayed hemorrhage during the healing process of a deep longitudinal ulcer that occurred at the onset of NOMI. It is not well-known how the ICG contrast pattern of the intestinal tract reflects the degree of ischemia. We considered this case to be suggestive, as the degree of ICG fluorescence reflected the degree of ischemia. The ileum, where the contrast effect of ICG was defective in the first surgery, had a deep longitudinal ulcer that bled during the second surgery, which would have been associated with severe mucosal necrosis at the time of the first surgery. In contrast, the cecum and ascending colon, which showed a fine granular ICG contrast pattern in the first operation, had scattered longitudinal ulcers in the second operation, suggesting mucosal necrosis in the first. Bulkley reported that measuring intestinal fluorescence intensity and pattern with intravenous fluorescein is useful in the assessment of intestinal ischemia [3]. We have revised and updated the table showing fluorescence patterns and intestinal viability from the Bulkely’s report and the present case (Table 1). Based on this case, we concluded that ‘Nonfluorescent lesions’ with obvious lack of fluorescence should be resected in the first surgery, while 'Patchy' and 'Perivascular' lesions are expected to be preserved but reassessed in the 2nd look surgery. Bowel resection should be performed only when deemed necessary. We believe that not removing the intestinal tract at the initial surgery was not an incorrect choice. If a segment of the intestinal tract was removed at the time of the initial surgery, there was a high probability that the patient would have had an ileal stoma.

**Table 1:** Patterns of ICG fluorescence

Pattern	Intensity	Texture	Intestinal mucosa	Outcome
Hyperemic	Increased	Uniform, smooth	Normal	Viable
Normal	Normal	Uniform, smooth	Normal	Viable
Fine granular	Normal to slightly decreased	Fine granular	Erosion	Viable
Patchy	Decreased	Patches of non-fluorescence ≥ 5 mm diameter	Ulcer	Nonviable or Viable
Perivascular	Decreased	Only perivascular areas stained	Necrosis/Deep ulcer	Nonviable > Viable
Nonfluorescent	None	None	Necrosis	Nonviable

This case suggests that the degree of ICG fluorescence in NOMI reflects the degree of intestinal ischemia. Intestinal tracts with decreased ICG fluorescence should be treated with the presence of mucosal necrosis and ischemia in mind, even if the serosa is viable. Therefore, it is important to treat with the possibility of necrosis and perforation in the acute phase, hemorrhage in the subacute phase, and stenosis in the chronic phase in mind. Therefore, frequent blood tests and CT should be performed during the acute phase to confirm the absence of intestinal perforation or necrosis; during the subacute phase, the possibility of bleeding should be considered, anemia and hematochezia progression should be checked, diet and anticoagulation therapy should be carefully monitored, and during the chronic phase, symptoms of intestinal obstruction due to stricture should be checked. In practice, perforation, necrosis, and stricture are easily identified on examination, but bleeding is difficult to predict, because it occurs suddenly.

In this patient, intraoperative ICG fluorescence showed patchy or perivascular patterns, but the patient recovered without bowel resection in the acute phase. However, these findings were indicative of severe intestinal ischemia, and the patient ultimately underwent bowel resection due to significant late complications. We believe that this patch pattern or perivascular pattern is not a finding that indicates that resection is not necessary, but rather a finding that should be given more consideration for second-look surgery as recommended by the American Gastroenterological Association guidelines [12, 13].

## Conclusions

Herein, we report a case of delayed bleeding after conservative treatment for NOMI. Delayed bleeding sites were defined as those in which the ICG blood flow was absent intraoperatively. Intraoperative ICG fluorescence imaging is useful for determining the degree of intestinal ischemia in NOMI. If the ischemic intestine with poor ICG contrast effect is not resected, late complications, such as postoperative bleeding and stenosis, should be noted.

## Data Availability

Not applicable.

## Abbreviations

CT: Computed tomography
ICG: Indocyanine green
NOMI: Non-obstructive intestinal ischemia
PGE1: Prostaglandin E1
SMA: Superior mesenteric artery
